# Integrative analysis of grapevine (*Vitis vinifera* L) transcriptome reveals regulatory network for Chardonnay quality formation

**DOI:** 10.3389/fnut.2023.1187842

**Published:** 2023-05-30

**Authors:** Guangqing Fu, Yanhua Ren, Jun Kang, Bo Wang, Junxiang Zhang, Jinggui Fang, Weimin Wu

**Affiliations:** ^1^Research Institute of Jiangsu Academy of Agricultural Sciences, Nanjing, Jiangsu, China; ^2^Department of Horticulture, Nanjing Agricultural University, Nanjing, Jiangsu, China; ^3^Horticultural College, Qingdao Agricultural University, Qingdao, Shandong, China; ^4^Food and Wine Academy, Ningxia University, Yinchuan, Ningxia, China

**Keywords:** wine grapes, fruit quality, transcriptome, weighted correlation network analysis, regulatory networks

## Abstract

Anthocyanins, total phenols, soluble sugar and fruit shape plays a significant role in determining the distinct fruit quality and customer preference. However, for the majority of fruit species, little is known about the transcriptomics and underlying regulatory networks that control the generation of overall quality during fruit growth and ripening. This study incorporated the quality-related transcriptome data from 6 ecological zones across 3 fruit development and maturity phases of Chardonnay cultivars. With the help of this dataset, we were able to build a complex regulatory network that may be used to identify important structural genes and transcription factors that control the anthocyanins, total phenols, soluble sugars and fruit shape in grapes. Overall, our findings set the groundwork to improve grape quality in addition to offering novel views on quality control during grape development and ripening.

## 1. Introduction

Fruit quality and flavor are crucial factors that influence consumer preference and market competitiveness. However, in the last fifty years, breeding efforts have mainly concentrated on enhancing fruit yield and disease resistance, rather than quality and flavor traits. As a result, commercially produced fruits have been perceived by consumers as lacking their distinctive quality and flavor (1, 2). Molecular breeding for quality-regulating genes may be a potential solution to restore lost quality flavors. However, accurately measuring quality flavor phenotypes can be challenging since they are heavily influenced by environmental factors and may not always be easily quantifiable (3). Chardonnay is mostly used to make white wine, which is mostly pale gold in color. The charm of Chardonnay lies in its changeable style and wide adaptability (4). The wines made from Chardonnay usually have the smell of pineapple, green apple or pear, it has a slightly sweet taste which is more suitable for Chinese tastes (5). However, the cultivation of Chardonnay grapevine is easily affected by a variety of external environments, cultivating high-quality, good-flavored Chardonnay grape is a prerequisite for making wines that taste good and are popular with consumers (6).

Grape fruit shape is an important appearance quality of grapes and is considered to be one of the main selection criteria for breeding (7). Consumers judge the yield, quality and nutritional value of fruits based on their size and shape. The shape of a fruit is commonly measured using the fruit shape index, which is the ratio of the longitudinal diameter of the fruit to its transverse diameter (8). Sugar is the basic substance for the growth and development of wine grape fruit, and the basic raw material for quality and flavor substances (9). Soluble sugar is an important factor of fruit quality, it includes fructose, glucose and sucrose in grape berries. The main function of sugar is to ferment into ethanol, which determines the alcohol content of wine. In addition, the formation of aroma substances is also related to sugar, it can form higher alcohols, esters and aldehydes in wine, making different wines have unique aroma characteristics (10).

Grape fruit contains a significant amount of phenolic compounds, including hydroxycinnamic acid and flavonoids. These compounds are considered secondary metabolites and play a crucial role in determining the quality of wine. Hydroxycinnamic acids, such as coumaric acid, caffeic acid, ferulic acid, cinadic acid and their derivatives, are known to accumulate in both grape skin and pulp (11). Grapes contain three major classes of flavonoids, namely proanthocyanidins, anthocyanins, and flavonols. Proanthocyanidins, also known as condensed tannins, are polymers of flavan-3-ol monomeric units like catechin, epicatechin and epicatechin-3-o-gallocatechin. These compounds are mainly found in grape skins and seeds. On the other hand, flavonols and anthocyanins are only detected in the berry skins. (12). All of these substances have crucial physiological roles in the growth of grape berries, including the scavenging of free radicals, pigmentation and co-pigmentation, UV radiation protection, and defense against fungi (13, 14). Additionally, their contributions to red wine color, bitterness, astringency and antioxidant properties have drawn a lot of attention in recent years in order to better understand the mechanisms that control phenolic biosynthesis and anthocyanin synthesis in grapes as well as their potential health benefits (15).

The metabolic dynamics and underlying regulatory mechanisms of high-quality tastes throughout grape development and ripening were examined in this study. We selected three growth stages (40 DAP, 80DAP and 120DAP) of Chardonnay cultivars from six ecoregions in Ningxia, China. We created a regulatory network that controls grape fruit shape, soluble sugars, total phenols and anthocyanins accumulation by fusing quality-related studies with transcriptome analysis. In addition, we identified proteases and transcription factors that regulated key pathways. This study laid the groundwork for the advancement of grape quality and offered a new concept for the regulation of wine grape quality.

## 2. Materials and methods

### 2.1. Plant material and measurements

Grapevine (*Vitis vinifera* L. Chardonnay) at different developmental stages were collected from six ecoregions in Ningxia Province, China. The six ecological zones are HJZ, ZHYS, CC, XG, XLS and HSB. Fruits were taken out between 40 days following the early flowering period (DAP) and 120 days, which included 80 days, at various developmental phases. And named them as the enlargement period (40 DAP), the color transition period (80 DAP) and the maturity period (120 DAP). Small chunks of the fruit’s flesh (without the skin or seeds) were sliced up and quickly frozen in liquid nitrogen before being kept at −80°C for additional transcriptome study. Every experiment was broken down into three biological replicates, with 15 uniformly sized fruits in each repeat.

Throughout fruit development and ripening, fruit weight, vertical and horizontal diameter and soluble solids content (SSC) were monitored. A drop of the mashed fruit’s supernatant was placed on a digital hand-held refractometer (Atago, Tokyo, Japan) to determine SSC. At each stage, measurements were taken using three biological duplicates.

### 2.2. Determination of berry total sugars, total phenols and anthocyanins components

Soluble sugars were analyzed according to the method described (16). A sample of 0.5 g of liquid nitrogen-pulped berry pulp was weighed, 1.5 mL of 80% ethanol was added, it was centrifuged at 12,000 rpm for 10 min, the supernatant was removed and filtered through a 0.22 μm aqueous filter head into a sample bottle for measurement. The chromatographic conditions for the determination of the soluble total sugar were as follows: Prevail Carbohydrate ES 5 μ column (100 mm × 4.6 mm, 5 μm); mobile phase: V (acetonitrile): V (water) = 80:20; column temperature: 50°C; flow rate: 1.0 mL/min; injection volume: 20 μL. The chromatographic conditions for the determination of the organic acids were as follows: column. Discovery C18 column (25 cm × 4.6 mm, 5 m); mobile phase was 50 mM K_2_HPO_4_ solution, pH adjusted to 2.4 with phosphoric acid; column temperature 30°C; flow rate 0.5 mL/min; injection volume 2  μL; detection wavelength was 210 nm. The sugars analyses were performed using three biological replicates.

Total phenolic were determined by HPLC (17). Weigh 1.0 g of the sample, grind the sample with liquid nitrogen in a centrifuge tube, use 10 mL of 80% methanol as the extraction solution. Then ultrasonic extraction is 20 min, extracted in a constant temperature water bath shaker at 25°C for 12 h, filtered with a 0.45 μm filter, and stored at −40°C for later use. The chromatographic conditions as follows: HPLC 1260 (Agilent, United States), Waters Xterra RP18 (100 mm × 4.6 mm, 3.5 μm) column, mobile phase A is water-formic acid (1,000: 2), mobile phase B is acetonitrile-A (80: 20), flow rate 0.6 ml· min^−1^, column temperature: 30°C, injection volume: 20 μL.

Total anthocyanins were determined by LC–MS (18). Weigh 1.0 g of berry peel with liquid nitrogen into a centrifuge tube, add 10 mL of 0.1% hydrochloric acid-methanol extract, ultrasonically extract for 20 min, centrifuge at 4°C for 15 min, and aspirate the supernatant. The liquid was filtered through a 0.22 mm organic column. The extracted anthocyanins were analyzed by LC–MS (G2-XS QT, Waters), chromatographic column: 2.1 × 100 mm ACQUITY UPLC BEH C18 column; flow rate was 0.4 mL/min; injection volume was 2 μL. Buffer A is 0.1% formic acid in water, and buffer B is 0.1% formic acid-acetonitrile solution. The total phenolic and total anthocyanins analysis was performed with three biological replicates.

### 2.3. RNA sequencing and data analysis

Frozen fruit was used to extract total RNA and the Illumina HiSeq-2000 platform was used to create mRNA libraries for each sample and sequence the results. After online sequencing, all relevant data was converted into raw data for data analysis. To acquire clean reads, low quality, linker contamination and reads with unknown base N content were removed. Clean reads were mapped to the *V. vinifera* reference genome^1^ by using HISAT2 (19). Transcripts per million (TPM) was determined after featureCounts was used to count the mapped fragments for each gene. Genes were deemed to be expressed if their averaged TPM had three replications. Using the DESeq software, differential expression genes (DEGs) were identified based on the counts of each transcript across libraries (20). A *p*-value <0.05 and | log_2_ (fold change) | > 1 were set as thresholds for significant differential expression. The cluster Profiler R package was used to perform gene ontology (GO) and Kyoto encyclopedia of genes and genomes (KEGG) pathway annotations with thresholds of FDR < 0.05 and P-value < 0.05, respectively (21). Functional annotation of all DEGs was also performed by using MapMan (Vvnifera 145)^2^ (22). The NCBI repository has the RNA-seq raw data from this paper under the accession number GSE231025.

### 2.4. WGCNA and gene network visualization

The weighted gene co-expression network analysis (WGCNA) tool in R was used to create co-expression network modules from DEGs after removing undetectable or relatively low expression genes (TPM < 10). The co-expression modules were obtained using automatic network construction function (blockwiseModules) with default parameters, apart from the soft threshold power of 12, TOMtype was signed, TOMsimilarity_Threshold was 0.485 and minModuleSize was 20. On eigengenes, the initial clusters were combined. Each module’s Eigengene value was determined and used to search for associations with the weight, vertical and horizontal diameters, concentration of soluble solids, soluble sugars, total phenolic and total anthocyanins produced during fruit development and ripening. The software Cytoscape and TBtools were used to visualize the networks and heat map (23, 24).

### 2.5. Real-time quantitative PCR analysis

Using a Plant RNA Purification Reagent (Invitrogen), total RNA from fruit at various growth and ripening phases was isolated in according with the manufacturer’s instructions. The DNase (TAKARA, Dalian, China) enzyme was used to degrade the genomic DNA. The Hifair II 1st Strand cDNA Synthesis SuperMix for RT-qPCR (YEASEN, Shanghai, China) was used to create the cDNA and SYBR Green (Vazyme, Nanjing, China) was used in the RT-qPCR amplification processes using an ABI 7500 Real-Time quantitative PCR System (Applied Biosystems, United States). The grape Actin gene was used as an internal control in the analysis of three biological replicates. The primers for RT-qPCR are listed in Supplementary Table S8.

## 3. Results

### 3.1. Berry quality components during fruit development and ripening of *Vitis vinifera* L. Chardonnay

**Figure 1:**
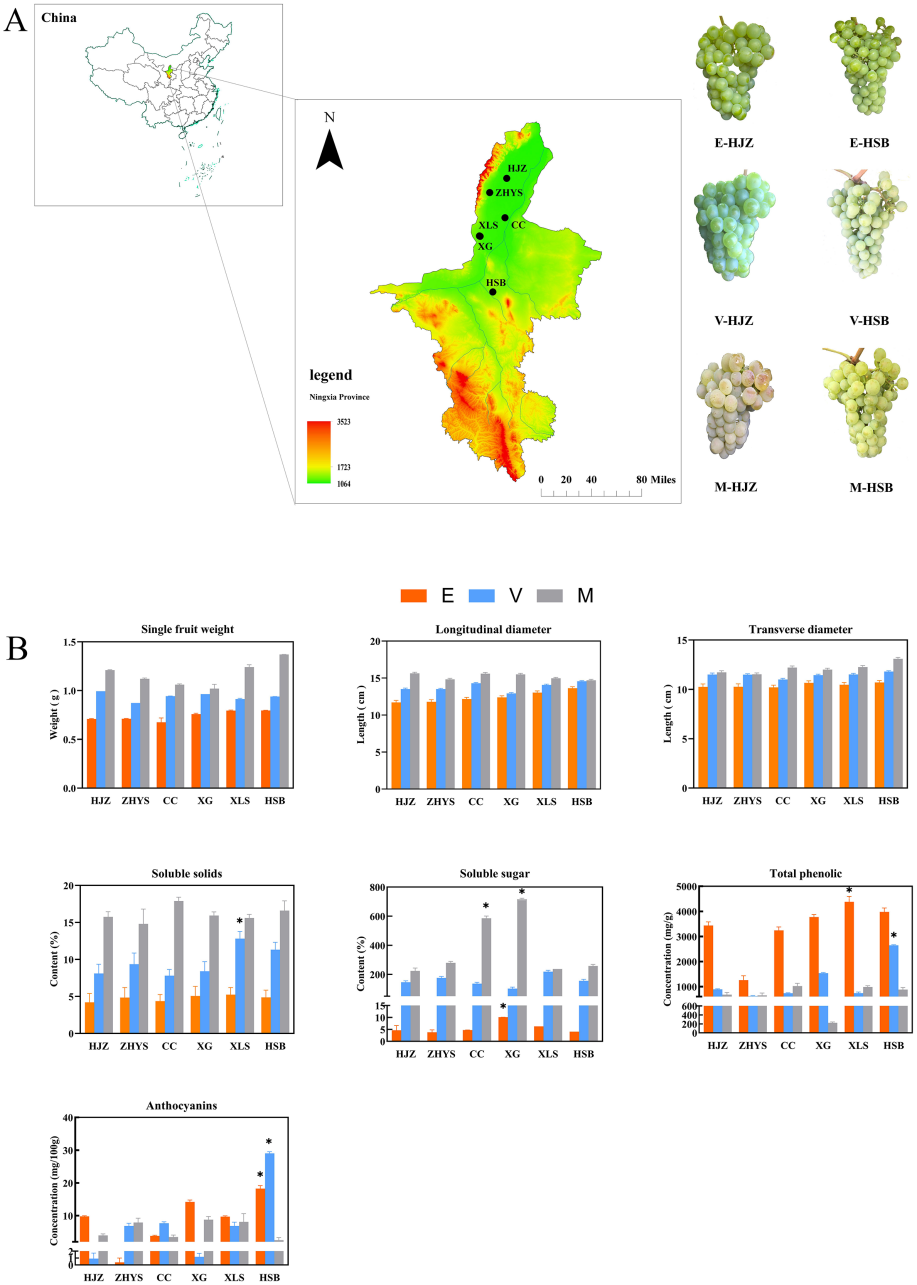
Three development stages of Chardonnay grape fruit were selected for analysis. **(A)** Grapevine (*Vitis vinifera* L. Chardonnay) at different developmental stages were collected from six ecoregions in Ningxia Province, China. **(B)** Single fruit weight, vertical and horizontal diameters, soluble solids, soluble sugars, total phenols and total anthocyanins were measured in three developmental stages of grape fruit. The values shown are mean ± standard deviation. E enlargement period. V color transition period. M maturity period.

To investigate the berry quality components-associated transcription regulatory network during grapevine different developmental processes, 3 stages including enlargement period (40 DAP), the color transition period (80 DAP) and the maturity period (120 DAP) of *Vitis vinifera* L. Chardonnay were selected from six ecoregions in Ningxia Province, China (Figure 1A; Supplementary Figure S1). In three stages, the six regions’ grape single fruit weight, fruit longitudinal diameter, fruit transverse diameter and soluble solids all gradually increased. The above measurement indicators are slightly different in different ecological zones, which may be related to the different environments (Figure 1B).

Fruit single-grain weight, fruit longitudinal diameter and transverse diameter are the main factors affecting grape shape and yield. In this experiment, we used measuring tools to measure the fruit single grain weight, longitudinal diameter and transverse diameter of Chardonnay grapes at three development and maturity stages in six different ecological zones. It was found that the change trends of the above three indicators in different ecological zones were consistent, and the specific performance was that the indicators of Chardonnay grapes were the smallest in the expansion period, the indicators in the color transition period were in the middle and the indicators in the maturity period were the largest (Figure 1B). The measurement indicators in different ecological zones are slightly different, which may be related to the different environmental climates in the regions.

The primary ingredients in grapes that give them their distinct flavor are soluble solids and soluble sugars. We employed a gas chromatography-mass spectrometry system (GC-MS) based metabolic analysis approach to track changes in soluble sugars using gas chromatography (GC) and a digital hand-held refractometer to measure soluble solids in order to examine flavor dynamics during grapevine development and ripening. It was found that the soluble solids did not change significantly during the enlargement and the color transition periods, but the content increased significantly during the maturity period, indicating that the accumulation of soluble solids in Chardonnay grape was completed in the maturity period. The change of soluble sugar content was significant in two of the six ecological zones (CC and XG). Compared with the enlargement period, the soluble sugar content showed a geometric multiple increase at the maturity period, especially in the two ecological zones CC and XG, which may be related to the climate environment of an ecological zone and artificial management (Figure 1B).

There are a lot of polyphenolic compounds in grapes. Wine undergoes fermentation, which increases the polyphenol content, stabilizes the composition and greatly boosts antioxidant potential. Grape anthocyanin content is frequently used as a significant factor to assess the quality of wine since it has a significant impact on grape color. In this experiment, total phenols content was generally higher at the enlargement period and lower at the maturity period, contrary to the trend of other physiological measures in this experiment (Figure 1B). The variation of anthocyanin content in the six ecological zones was irregular, it is noteworthy that in the HSB ecological zone, the variation was significant during the enlargement and color change periods, which may be related to the fact that the anthocyanin content of grapes is highly susceptible to temperature, light, and phytohormones.

### 3.2. Genetic basis of dynamic changes during fruit development and ripening of *Vitis vinifera* L. Chardonnay

RNA-seq data were produced for 18 different developmental and ripening stages in order to examine the genetic basis of grape development and ripening. 6.65 Gb clean reads were produced on average after adaptor reads, unclear reads and low-quality reads were eliminated. The grapevine reference genome was then mapped by more than 85.66% of high-quality reads from various sample types, of which 82.12% or more were mapped uniquely genome, of which 82.12% or more were mapped uniquely (Supplementary Table S1). Transcripts per million (TPM) readings’ indications of expression levels revealed significant interactions between biological replicates. Transcriptional values greater than 1 were present for more than half of the genes. The level of sample repeatability was assessed in line with the R2 value in order to examine the accuracy of the data and get a general idea of variances across all samples. TPM measurements were also used in the principal components analysis (PCA). Principal component (PC) 1 accounted for 57.6% of the variance and PC2 for 33.1% of it, according to the combined analysis of variance, which identified 90.7% of the variance among the 18 groups (54 samples). Overall, the information provided demonstrated that there was little to no variation among all sample replications and that high-quality sequencing data and biological duplicates are highly reproducible (Supplementary Figure S2; Supplementary Table S2). In total, 25,133 genes were found to be expressed in 18 different developmental and ripening stages (Supplementary Table S3).

**Figure 2:**
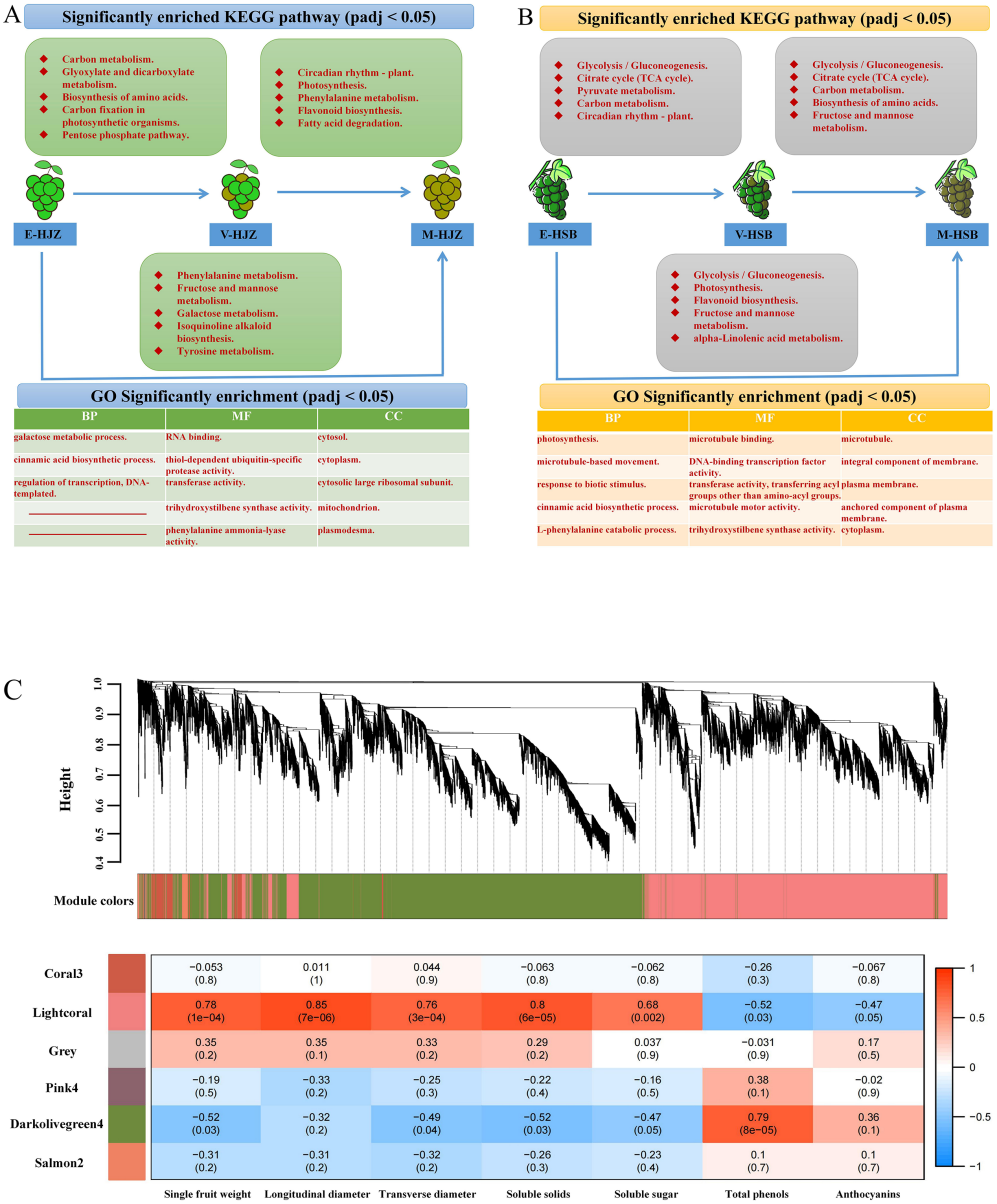
Transcriptome analysis of three periods of grapevine fruit development and maturity. **(A,B)** Different ecological regions were significantly enriched in the top 5 KEGG vocabulary and at least two top 5 GOs in the three developmental periods. **(C)** A dendrogram of co-expression modules (clusters) identified by WGCNA at fruit development and ripening stages. The main branches make up 6 modules of different colors. Additionally, a heatmap of the physiological shape correlation of the modules is shown. Each row corresponds to a module represented by a different color. Each column corresponds to a physiological index. Red indicates that clusters are positively correlated with tissue. Blue indicates a negative correlation.

Figures 2A,B and Supplementary Figure S3 show a number of up- and down-regulated DEGs identified at two adjacent periods during enlargement, color transition and maturity responses in six ecoregions. Notably, ‘carbon metabolism’, ‘glycolysis / gluconeogenesis’, ‘circadian rhythm - plant’, ‘biosynthesis of amino acids’, ‘carbon fixation in photosynthetic organisms’ and ‘citrate cycle (TCA cycle)’ were the main pathways functioning during enlargement – color transition periods. ‘circadian rhythm - plant’, ‘photosynthesis’, ‘glycolysis/gluconeogenesis’ and ‘biosynthesis of amino acids’ were the main pathways during color transition and maturity periods. Besides, ‘galactose metabolism’, ‘flavonoid biosynthesis’, ‘phenylalanine metabolism’, ‘fructose and mannose metabolism’, ‘carbon fixation in photosynthetic organisms’ and other photosynthesis-related pathways were enriched GO and KEGG terms that were worthy of attention in the transcriptome (enlargement and maturity periods) (Supplementary Tables S4, S5).

### 3.3. Generation of transcriptional metabolic regulatory network

To gain further insight into the regulation of the transcriptional metabolism changes throughout grapevine development and ripening, WGCNA was performed to investigate the co-expression networks of DEGs. After filtering, in total 18,847 genes were used for further analysis and co-expression network in grapevine cultivars (Supplementary Table S6). The highest connectivity is achieved at a power value equal to 12 (Supplementary Figure S4). A total of 6 co-expression modules were identified based on their similar expression patterns. The module-trait correlation heat map showed that the accumulation of lightcoral module transcripts was significantly positively correlated with the shape and flavor changes of grape, including fruit single-grain weight, longitudinal diameter, transverse diameter, soluble solids and soluble sugar. The darkolivegreen4 module was associated with the above factor significant negative correlation. In contrast, the accumulation of transcripts in the darkolivegreen4 module was significantly positively correlated with grape total phenolics and anthocyanins. The lightcoral module was significantly negatively correlated with total phenolics and anthocyanins (Figure 2C).

### 3.4. Mining for differentially expressed genes in grape fruit shape regulation and flavour networks

**Figure 3:**
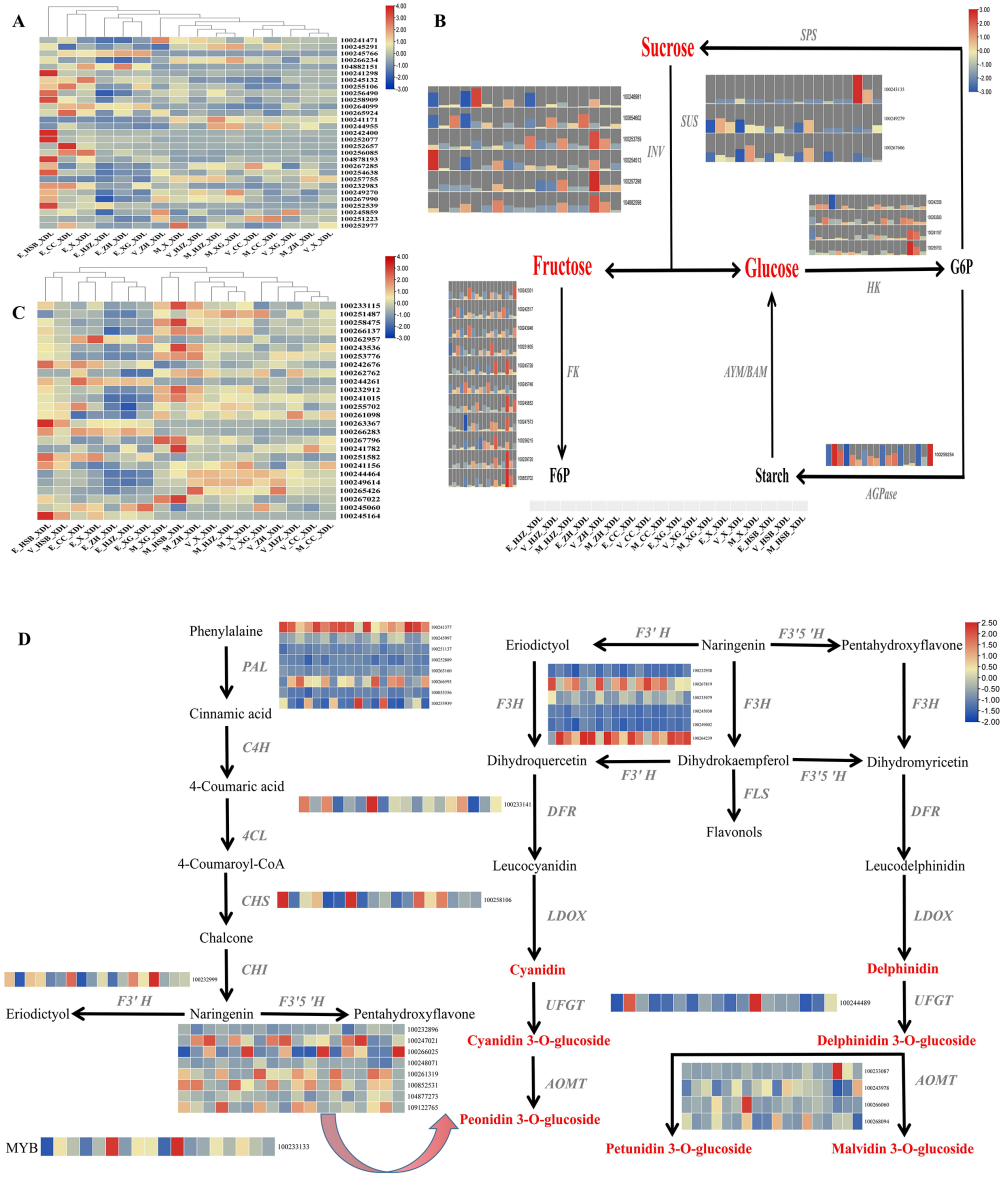
The key regulatory network for changes in morphology, color and flavor transcripts during grapevine development and ripening. **(A)** Heat map of key genes regulating grape shape. **(B)** Metabolic pathways of soluble sugars and expression patterns of involved genes. INV, invertase; SUS, sucrose synthase; HK, hexokinase; AMY/BMY, α−/β-amylases; FK, fructokinase; AGPase, ADP glucose pyrophosphorylase; SPS, sucrose phosphate synthetase. **(C)** Heat map of transcriptional expression of genes regulating grape phenolic compounds. **(D)** Gene expression patterns of the anthocyanin biosynthetic pathway in grape. PAL, Phenylalanin ammonia-lyase; C4H, Cinnamate-4-hydroxylase; 4CL, 4-coumarate: coenzyme A ligase; CHS, Chalcone synthase; CHI, Chalcone isomerase; F3H, Flavanone3-hydroxylase; F3’H, Flavanone 3′-hydroxylase; F3’5’H, Flavanone 3’5’-hydroxylase; DFR, Dihydroflavonol 4-reductase; FLS, Flavonol synthase; LDOX, Leucoanthocyanin dioxygenase; UFGT, UDP glucose:flavonoid 3-*O*-glucosyltransferase; AOMT, *O*-methyltransferase.

While consumers are satisfied with the traditional supply of fruit, they are turning to new, exotic and high-quality products (25). It is therefore particularly important to produce excellent varieties with beautiful appearance, high nutrition and high efficiency (26). The shape of the grape fruit is one of the most important factors in consumer selection. We have identified a number of important DEGs in grape shape in the lightcoral module and darkolivegreen4 module, including 12 IQ-DOMAIN proteins, 7 MADS-box transcription factors, 3 indole-3-pyruvate monooxygenases, 2 EIN3-binding F-box proteins and ethylene receptors and one myb-related protein and brassinosteroid-6-oxidase (Supplementary Table S7). Then, the heat map enrichment of the above differential genes was carried out and it was found that the high expression was mainly concentrated in the grapevine enlargement periods, there was no expression or weak expression in the color transition and maturity periods (Figure 3A).

Soluble sugars are among the most important components contributing to the characteristic flavor of grape, it includes sucrose, fructose and glucose. Interestingly, the accumulation of soluble sugars was highly correlated with the lightcoral and darkolivegreen4 modules. To generate the regulatory network associated with soluble sugars metabolism, we examined the structural genes involved in soluble sugar metabolic pathway identified in the lightcoral and darkolivegreen4 modules. We identified 25 soluble sugar-metabolizing genes including 6 invertases (INV), 3 sucrose synthases (SUS), 4 hexokinases (HK), 10 fructokinases (FK) and 1 ADP glucose pyrophosphorylase in the lightcoral and darkolivegreen4 modules (Supplementary Table S7) whose expression was highly correlated with the accumulation of the soluble sugars. Most of the genes were highly expressed in the grapevine enlargement and the color transition periods, but lower in the maturity period (Figure 3B).

### 3.5. Production of grape phenolics and anthocyanins regulatory network

Chardonnay grapes are one of the main sources of wine grape varieties. Phenolic substances are crucial to the color and astringency of wine. They can endow grapes and wine with stable and high-quality color, full and rich taste, which is also the key to the health function of wine (27). Therefore, it is very meaningful to explore the changes of total phenols in grape. The phenolic acids in grapes are mainly derived from intermediate products of the shikimate pathway, other phenolics are produced from the end product of the shikimate pathway L-Phenylalanine via the phenylpropanoid biosynthetic pathway and the flavonoid biosynthetic pathway. In this study, we examined key structural genes related to the above-mentioned phenolic substance production pathway in the lightcoral and darkolivegreen4 modules, including 3-deoxy-D-arabino-heptulosonate-7-phosphate synthase (DAHPS), 3-dehydroquinate synthase (DHQS), quinate dehydrogenase (QDH)，shikimate kinase (SK)，5-enolpyruvylshikimate-3-phosphate synthase (EPSPS), chorismate synthase (CS), anthranilate synthase (AS) and prephenate dehydratase (PDT) (Supplementary Table S7). These structural genes showed different expression patterns during the development and ripening of grapevine, with generally high expression in the maturity period and low expression in the enlargement period (Figure 3C).

For wine grapes, anthocyanin content is often used as an important criterion for evaluating its quality (28). Since flavonoids and phenylalanine compounds are the main contributors to grape anthocyanins (29), we tried to generate regulatory networks for the metabolic pathways of the two compounds, screened the key structural genes involved in the above metabolic pathways in the lightcoral and darkolivegreen4 modules and found 8 phenylalanine ammonia-lyases (PAL), 1 chalcone synthase (CHS), 6 flavanone 3-hydroxylases (F3H), 1 flavonoid 3′ hydroxylase (F3’H), 8 flavonoid-3,5′-hydroxylases (F3’5’H), 1 dihydroflavonol 4-reductase (DFR), 1 UDP-sugar flavonoid glycosyltransferase (UFGT) and 4 caffeoyl-CoA *O*-methyltransferases (AOMT). At the same time, in network regulation, we also found a transcription factor MYB, which plays an important role in the regulation of anthocyanins (Supplementary Table S7). Among the 8 PALs, one gene was generally highly expressed in all periods of Chardonnay grapes, two genes were highly expressed in grapevine maturity period and weakly expressed in the color transition and enlargement periods. Among the 8 F3’5’Hs, 5 genes were highly expressed in the grapevine color transition and maturity periods and 3 genes were generally low expressed in the three grapevine development periods. Among the 6 F3’Hs, 2 genes were highly expressed in most periods. Four caffeoyl-CoA *O*-methyltransferases were weakly expressed or not expressed in the three developmental periods of grapevine (Figure 3D).

### 3.6. RT-qPCR validation

For RT-qPCR investigation, 10 DEGs were randomly chosen in order to validate the RNA-Seq results Supplementary Table S8. gene-specific RT-qPCR primer pairs are listed. The RT-qPCR and RNA-Seq data at various phases of development and maturation had similar expression patterns, according to the expression results, indicating the reliability of the RNA-Seq expression data.

**Figure 4:**
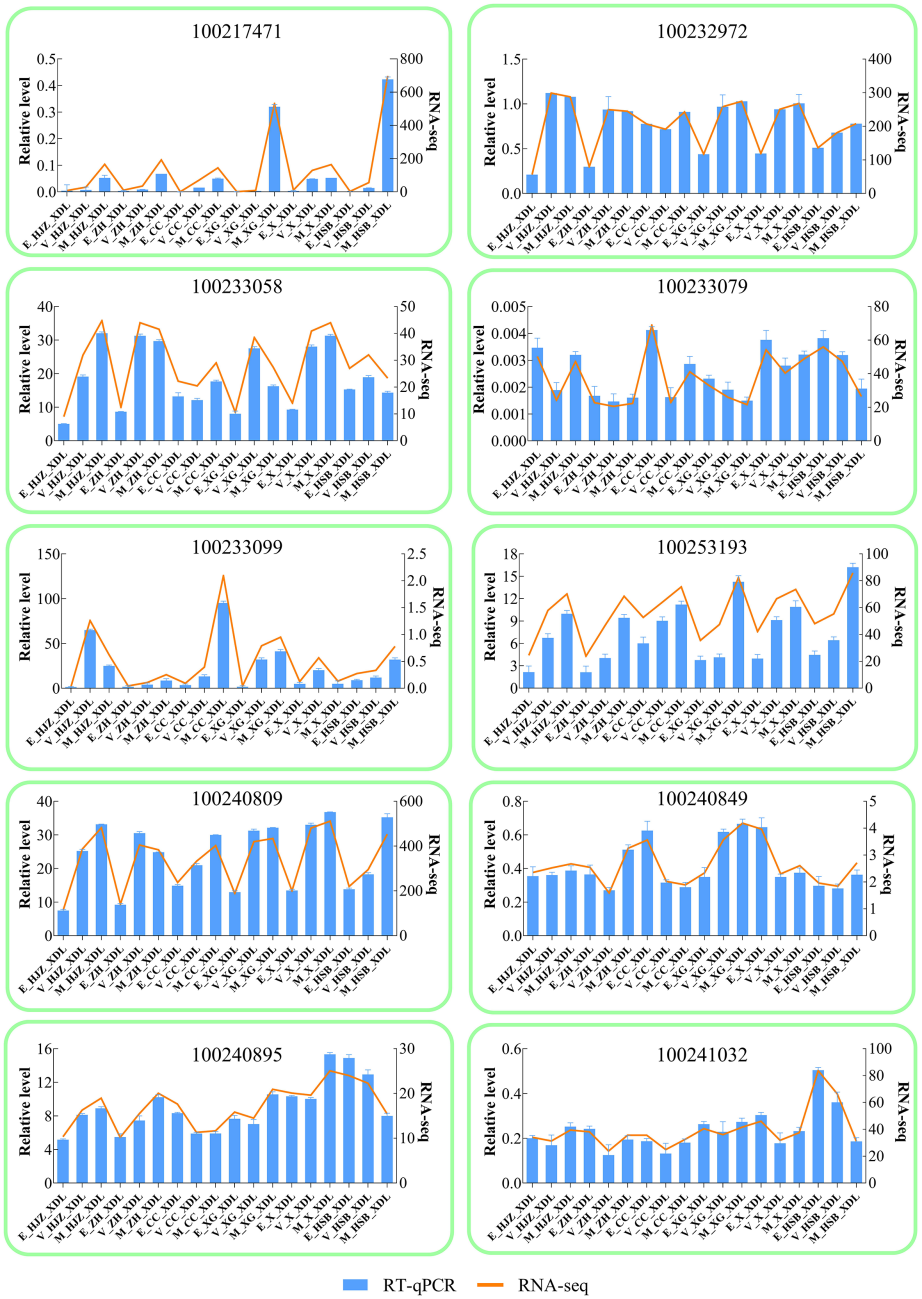
Validation of RNA-seq by RT -qPCR. The column chart and the main longitudinal coordinate represent the relative expression of quantitative real-time PCR (RT -qPCR), while the broken line diagram and the secondary longitudinal coordinate represent the TPM value of RNA-seq.

## 4. Discussion

Originally from Burgundy, France, the Chardonnay grape is a world-renowned variety for making excellent white wines. It has been introduced to China from France since 1979 and has subsequently started to be widely promoted and planted in China (30). The taste and quality of the grapes have a significant impact on the wine’s quality. In our study, Chardonnay grapes were collected from six different ecological zones in Ningxia Province, China. we monitor their growth and development and measure physiological indicators such as single fruit weight, longitudinal diameter, transverse diameter, soluble sugars, total phenols and total anthocyanins at the enlargement, color transition and maturity periods. In addition, transcriptome sequencing is also an important tool to monitor gene expression during different growth and developmental stages of Chardonnay grapes. We sequenced the transcriptomes of grapevine at different growth and developmental stages and correlated phenotypic traits to identify structural genes and transcription factor that regulate the growth and development of grapevine.

### 4.1. Chardonnay regulation of fruit shape genes in Chardonnay grapes

Fruit shape is an important appearance quality of grapes. Due to different preferences of consumers, fruit shape greatly affects people’s choice of grapevine varieties (31). Economically important traits such as yield, quality and nutritional value are judged on the basis of fruit size and shape (8). The genotype and environmental factors play a major role in determining grape shape, although growing practices also play a role. In our study, seven classes of key structural genes and transcription factors affecting grape shape development were identified (12 IQ-DOMAIN proteins, 7 MADS-box transcription factors, 3 indole-3-pyruvate monooxygenases, 2 EIN3-binding F-box proteins and ethylene receptors, one myb-related protein and brassinosteroid-6-oxidase) (Supplementary Table S7; Figure 3A). Scaffold proteins interact or combine with various proteins at specific locations such as cell membrane, cytoplasmic matrix or nucleus to form anchoring complexes, play an important role in signal transduction (32, 33). As scaffolding proteins, IQDs play important roles in plant growth and development (34). Wu et al. (35) reported that SUN/IQD can regulate cell division to prolong tomato growth, in IQD5-1 mutants, microtubule stability is reduced, resulting in disordered microtubules in cotyledon cells and reduced staggering in squamous cells. IQD5 plays a crucial role in regulating Arabidopsis leaf morphogenesis (36). MADS-box protein widely exists in eukaryotes and is an important class of transcription factors (37). Its gene is involved in the regulation of plant growth and development from root to flower and fruit (38). Dong et al. (39) showed that tomato with SlMADS1 gene silenced at fruit ripening, the amount of ethylene produced during the process is about 2 to 4 times that of the wild type. Terol et al. (40) found a MADS-box gene in Citrus clementina that may be involved in fruit regulation, specifically, the expression level in early-maturing varieties is lower than that in late-maturing varieties. In addition, cell growth is regulated by hormones such as auxin, gibberellin and brassinosteroids (41–43). Brassinolide can also change the shape of the fruit (44). Studies have shown that GW5 plays a role in the brassinosteroid signaling pathway to regulate the grain width and weight of rice (45); BR level changes have an effect on ovary growth and cell division in the early stage of cucumber fruit development (46). Interestingly, we found that IQD protein, MADS-box protein and brassinosteroid-6-oxidase were significantly expressed during the enlargement period of Chardonnay grapes and weakly expressed or not expressed during the color transition and maturity periods (Figure 3A). Therefore, we hypothesized that the shaping of fruit shape is mainly completed during the enlargement period during the growth of Chardonnay grapevine.

### 4.2. Regulatory network of sugar metabolism in Chardonnay grape

Sugar is closely related to fruit yield and quality. Different types of soluble sugar have different sweetness and the difference in soluble sugar content and ratio has a decisive impact on taste characteristics and sensory quality (47–49). Sugar metabolism is the center of the whole biological metabolism of plants, plants have evolved to form a complete sucrose–sucrose (Suc-Suc) cycle metabolism system, which regulates the utilization, storage and homeostasis of intracellular sugar (50). The sugar metabolism regulation network is complex and involves many enzymes and transporters (51). The improvement of fruit sweetness quality has always been an important goal in fruit breeding. It is important to investigate the genetic control mechanism of sugar content in fruits and to explore the molecular markers and key genes for fruit sweetness quality breeding (52). Sucrose can produce fructose and glucose under the action of invertase and sucrose synthase. In our study, most of invertase and sucrose synthase were highly expressed during the enlargement period, which indicated that the accumulation of sugar in grapevine mainly occurred in Early growth and development (Supplementary Table S7; Figure 3B). Interestingly, Xu et al. (53) reported that insertion of a transposon in the rice vacuolar invertase gene OsVIN2 resulted in the emergence of a small-grain mutant sgs1 with increased sucrose content and decreased hexose levels in sgs1 (54). Cassava MeCWINV3 regulates sugar partitioning from source to sink and affects storage root yield by maintaining sugar balance in cassava (55). The activity of sucrose synthase was higher in the early stage of grapevine development and its activity decreased in ripening (56). Islam et al. (57) identified six sucrose synthases in citrus, which play an important role in regulating sugar accumulation in fruits. Fructose and glucose produce fructose-6-phosphate (F6P) and glucose-6-phosphate (G6P) under the action of fructokinase and hexokinase, respectively. Yang et al. (58) showed that MdFRK2 is the key gene for fructose phosphorylation in apples and the homeostasis and signaling of fructose content can regulate sugar metabolism and content. Transient overexpression of FpHXK1 and its kinase-deficient mutants had different effects on the contents of glucose, sucrose, anthocyanins and total phenols in strawberry fruit (59). F6P can re-synthesize sucrose under the catalysis of sucrose phosphate synthase (SPS). McIntyre et al. (60) verified that the SPS gene can improve the ability of sucrose synthesis through expression in sugarcane. Overall, we monitored the sugar metabolism network during the growth and development of Chardonnay grapevine, in which various enzymes and transporters play important roles.

### 4.3. Regulatory network of total phenols in Chardonnay grape

Grape phenolics are widely present in the fruit, leaf and branch tissues and are the main components that influence the skin, the colour of the wine and the backbone of the wine (61). There are a large number of phenolic substances in grape, which are closely related to grape postharvest transportation and storage, preservation, disease resistance, wine color, flavor and other quality indicators (62). Phenolic substances are one of the important secondary metabolites in plants. A variety of metabolic processes in plants involve the synthesis of phenolic substances, among which the shikimic acid pathway is the primary pathway for the synthesis of phenolic substances (63, 64). In our study, the regulation of proteases related to the shikimate metabolic pathway was our focus and eight regulatory proteases were found, including DAHPS, DHQS, QDH, SK, EPSPS, CS, AS and PDT. The cluster enrichment of 8 related proteases found that they were mainly expressed significantly during the grapevine maturity period (Supplementary Table S7; Figure 3C). DAHPS is the initial enzyme of the shikimate synthesis pathway, catalyzing the synthesis of 3-deoxy-arabinoheptulose-7-phosphate from phosphoenolpyruvate and erythrose-4-phosphate (65). In Anji white tea, the expression levels of CsDAHPS gene also differed significantly at different developmental stages of the leaves, indicating that the gene responds to the whitening phenomenon in Anji white tea, while the expression of CsDAHPS gene enables tea trees to resist pathogenic infestation, improving the resistance of tea trees to stress (66). SK was able to catalyse the conversion of mangiferous acid to phosphoric acid-3-mangiferous acid. The expression of OsSK1 and OsSK3 was specifically increased at the spike tassel stage compared to pre- and post-tassel expression, suggesting that SK may influence floral organ development by controlling the mangiferic acid metabolic pathway (67). EPSPS mediates the synthesis of aromatic amino acids and some secondary metabolites in the mangiferous acid pathway. Overexpression of EPSPS increased stress resistance in *Arabidopsis thaliana* (68). In summary, the eight proteases not only affect the formation of grape phenols, but may also play an important role in plant stress resistance and floral organ development.

### 4.4. Regulatory network of anthocyanins in Chardonnay grape

As the largest branch of the flavonoid family, anthocyanins affect the color, taste nutritional value of wine and directly determine the color of grapes (23). Anthocyanins and tannins combine to form anthocyanin-tannin complexes, which soften the tannins in the wine, reduce its bitterness and roughness and make the taste more mellow. Anthocyanins can also scavenge free radicals, and their antioxidant activity is higher than that of vitamin C and vitamin E (69). In addition, anthocyanins have high anti-mutation, anti-cancer and anti-hyperglycemic activities, which endow wine with high nutritional value. The anthocyanins in wine grapes are a kind of flavonoid polyphenolic compounds with C6-C3-C6 as the basic skeleton, which are combined with sugar by glycosidic bonds (70, 71). Its biosynthesis goes through the synthetic pathway of phenylpropanoids and flavonoids (72). In our study, the ‘flavonoid biosynthesis’, ‘phenylalanine metabolism’ pathways were significantly enriched in Chardonnay grapes during the enlargement and the maturity periods (Figure 2A; Supplementary Figure S3). We focused on monitoring the biosynthesis pathway of phenylalanine and flavonoids, and found that a variety of proteases participated in the regulation of anthocyanin synthesis, including 8 PALs, 1 CHS, 6 F3Hs, 1 F3’H, 8 F3’ 5’Hs, 1 DFR, 1 UFGT and 4 AOMTs (Supplementary Table S7; Figure 3D). PAL is the key rate-limiting enzyme in the metabolism of phenylpropanoids. Xu et al. (73) treated grapes with N_2_O and found that the up-regulated expression of 12 PAL genes enhanced the metabolic activity of phenylalanine in grape, thereby increasing the anthocyanin content of grapes. Chen et al. (74) found that the tea tree CsPAL4 gene was preferentially expressed in young leaves and buds. Through the correlation analysis of anthocyanin components in purple leaf tea, it was found that CsPAL4 was closely related to the accumulation of different anthocyanins. F3’H and F3’5’H can catalyze the hydroxylation of dihydrokaempferol to dihydroquercetin and dihydromyricetin (75). Robinson et al. (76) used F3’H and F3’5’H gene silenced transgenic grapes to study the effects of these two genes on grape anthocyanins and the results showed that the decrease in the expression of a single gene in the two did not change the anthocyanin content, but led to changes in the composition ratio of anthocyanins. AOMT catalyzes the monoglycoside methylation of anthocyanins and delphinidins to produce glycosides of methylanthocyanidins, methyldelphinidins and delphinidins (77). DFR reduces dihydroflavonols to corresponding colorless anthocyanins (76). During fruit development, CHS is mainly distributed in the pericarp and vascular bundles, it is located in the cytoplasm, nucleus, cell wall, chloroplast, chromoplast and rough endoplasmic reticulum (78). The increase of CHS activity was consistent with the accumulation process of flavonoid content (79). In addition, the anthocyanin metabolic network is also regulated by regulatory factors such as MYB. MYB-related genes play a crucial role in the regulation of grape anthocyanin biosynthesis, mainly regulating the expression of 3GT (80). In our study, a MYB transcription factor was significantly expressed during Chardonnay enlargement period (Figure 3D). These results suggest that the related protease and transcription factor MYB in the phenylalanine and flavonoid biosynthetic pathway may regulate the production of grape anthocyanins.

## 5. Conclusion

Here, based on our transcriptome database, we constructed the differential expression of Chardonnay grapevine cultivars at various growth and developmental phases in six ecological zones of Ningxia, China. We monitor its growth and development and measure physiological indicators such as swelling stage, discoloration stage, single fruit weight at maturity stage, longitudinal diameter, transverse diameter, soluble sugar, total phenols and total anthocyanins. Based on WGCNA correlative physiological data, we focused on Chardonnay grape shape changes, soluble sugar synthesis, total phenolic metabolic network and anthocyanin synthesis. The shikimic acid metabolic pathway, carbon skeleton biosynthesis, cell wall synthesis, phenylalanine metabolism, flavonoid biosynthesis and other reaction pathways were enriched in different growth stages of grapevine. We excavated some structural genes and transcription factors that may focus on regulating grape shape development, soluble sugar synthesis, total phenol metabolism network and anthocyanin synthesis network, which will provide a basis for the next step to monitor grape quality at the molecular level.

## Data availability statement

The original contributions presented in the study are publicly available. This data can be found here: NCBI, GSE231025.

## Author contributions

WW and JF: conceived and designed the experiments. GF, YR, JK, and BW: analyzed the data. JK, YR, and GF: conducted the experiments and wrote the paper. JF, JZ, and WW: revised the paper. All authors contributed to the article and approved the submitted version.

## Funding

This work was supported by the General Project of National Natural Science Foundation of China (32272647), Key R&D Program of Shandong Province (2022LZGCQY1018), High-level Scientific Research Foundation of Qingdao Agricultural University (665/1119002) and Key R&D Program of Jiangsu Province (BE2022381).

## Conflict of interest

The authors declare that the research was conducted in the absence of any commercial or financial relationships that could be construed as a potential conflict of interest.

## Publisher’s note

All claims expressed in this article are solely those of the authors and do not necessarily represent those of their affiliated organizations, or those of the publisher, the editors and the reviewers. Any product that may be evaluated in this article, or claim that may be made by its manufacturer, is not guaranteed or endorsed by the publisher.

## Footnotes

^1^ ftp://ftp.ensemblgenomes.org/pub/release-23/plants/gtf/vitis_vinifera/

^2^ http://mapman.gabipd.org/web/guest/mapmanstore
