# Supplementary material for: Integrative analysis of grapevine (Vitis vinifera L) transcriptome reveals regulatory network for Chardonnay quality formation
Source: Front Nutr. 2023 May 30;10:1187842. doi: 10.3389/fnut.2023.1187842 (PMC10265639; doi:10.3389/fnut.2023.1187842)
Supplement: Supplementary file 2 [file Data_Sheet_1.PDF]

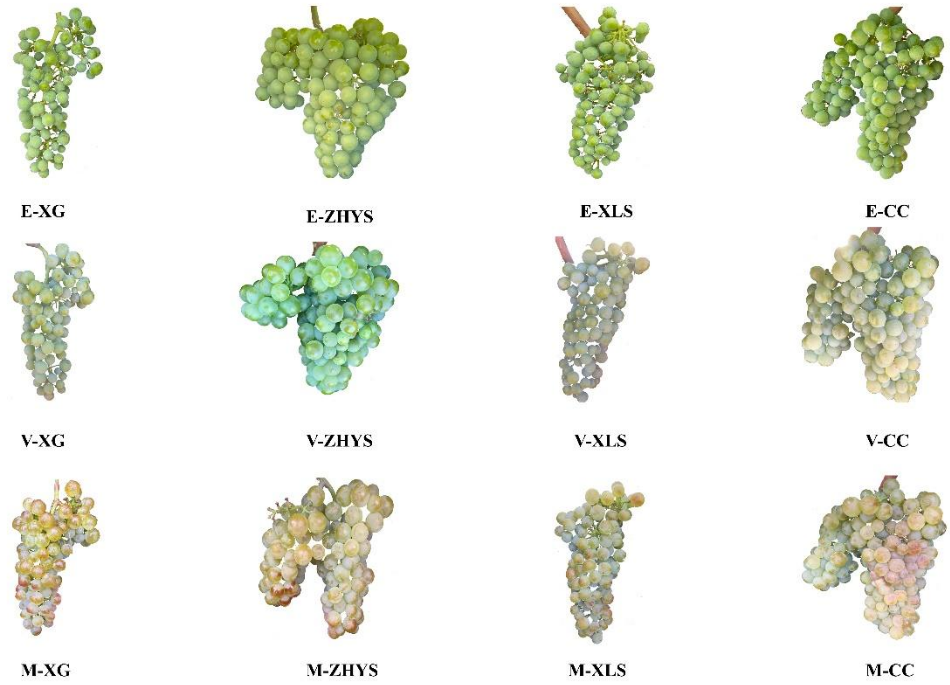

Figure S1 Displays of Chardonnay grapes at different stages of development were collected in the other four different ecoregions.

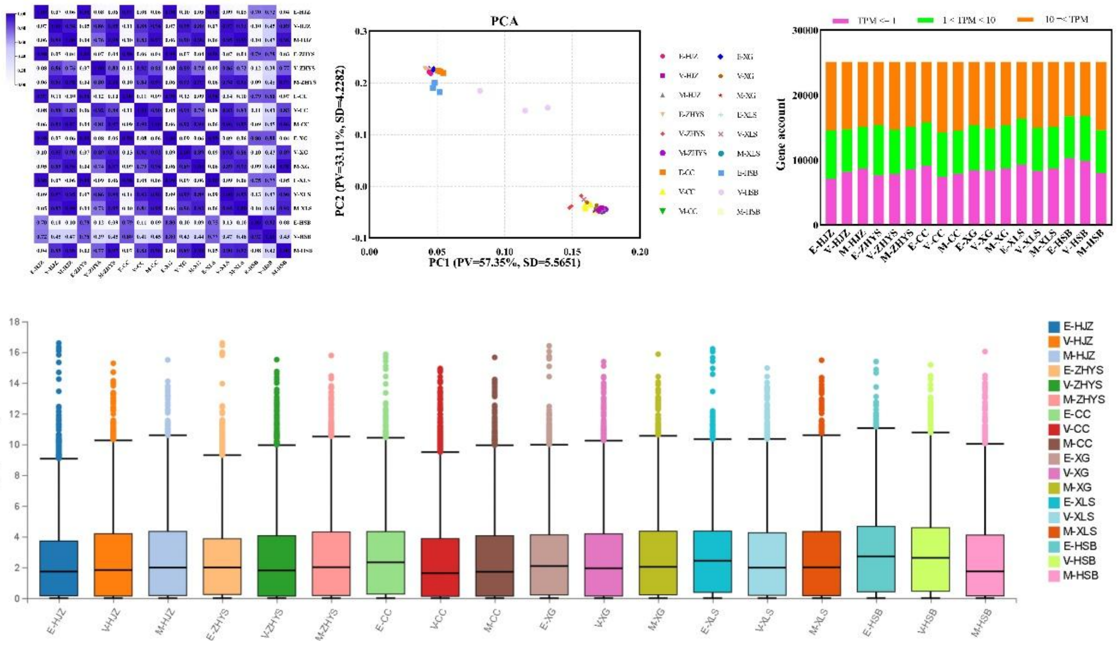

Figure S2 Different sample analysis and gene expression information. These include the expressed gene rate based on all genes, the number of genes with different fragments per kilobase transcripts per million (TPM) value, correlation matrix showing the correlation between samples and Principal Component Analysis (PCA) were performed on the biological replicates of each sample.

	E-HJZ	V-HJZ	M-HJZ	E-ZHYS	V-ZHYS	M-ZHYS
E-HJZ	—	6802↑ 7311↓	—	—	—	—
V-HJZ	—	—	2818↑ 3070↓	—	—	—
M-HJZ	3840↑ 4559↓	—	—	—	—	—
E-ZHYS	—	—	—	—	5622↑ 6061↓	—
V-ZHYS	—	—	—	—	—	3978↑ 4183↓
M-ZHYS	—	—	—	3219↑ 4721↓	—	—

	E-CC	V-CC	M-CC	E-XG	V-XG	M-XG
E-CC	—	6424↑ 6649↓	—	—	—	—
V-CC	—	—	2648↑ 2904↓	—	—	—
M-CC	3545↑ 4452↓	—	—	—	—	—
E-XG	—	—	—	—	6014↑ 6269↓	—
V-XG	—	—	—	—	—	5880↑ 5694↓
M-XG	—	—	—	3886↑ 4597↓	—	—

	E-XLS	V-XLS	M-XLS	E-HSB	V-HSB	M-HSB
E-XLS	—	4757↑ 4943↓	—	—	—	—
V-XLS	—	—	2697↑ 4348↓	—	—	—
M-XLS	2960↑ 4974↓	—	—	—	—	—
E-HSB	—	—	—	—	3353↑ 2150↓	—
V-HSB	—	—	—	—	—	6488↑ 7523↓
M-HSB	—	—	—	4445↑ 5868↓	—	—

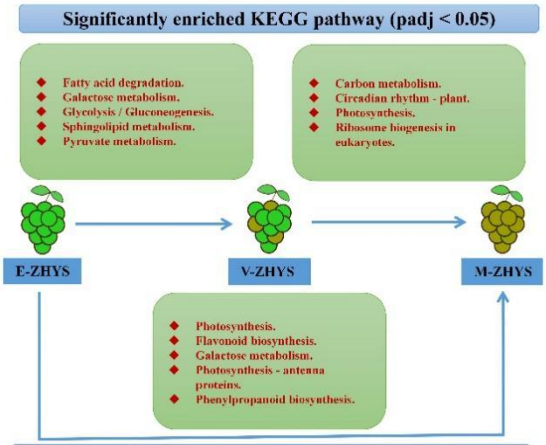

GO Significantly enrichment (padj < 0.05)

BP	MF	CC
photosynthesis, light harvesting in photosystem I.	trihydroxystilbene synthase activity.	chloroplast thylakoid membrane.
protein-chromophore linkage.	phenylalanine ammonia-lyase activity.	nucleolus.
photosynthesis, light harvesting.	ammonia-lyase activity.	integral component of membrane.
photosynthesis.	DNA-binding transcription factor activity.	plasma membrane.
aromatic amino acid family biosynthetic process.	transferase activity, transferring acyl groups other than amino-acyl groups.	photosystem I.

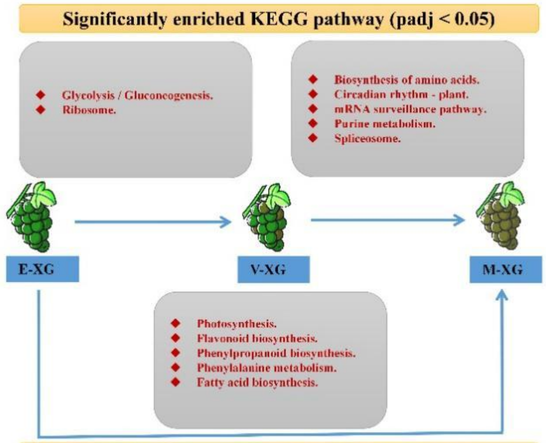

GO Significantly enrichment (padj < 0.05)

BP	MF	CC
photosynthesis.	phenylalanine ammonia-lyase activity.	cytosol.
cinnamic acid biosynthetic process.	ammonia-lyase activity.	cytoplasm.
response to biotic stimulus.	RNA binding.	photosystem II.
intracellular signal transduction.	mRNA binding.	integral component of membrane.
mRNA cis splicing, via spliceosome.	calmodulin binding.	plasma membrane.

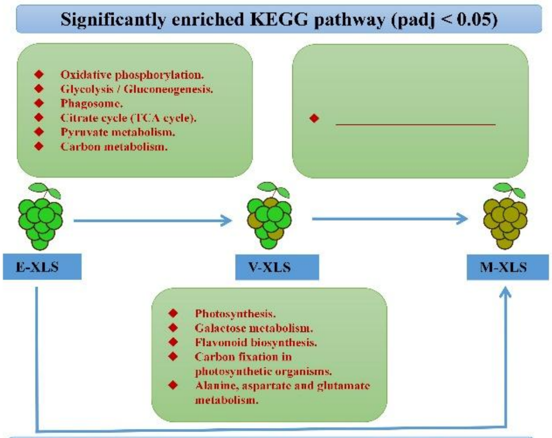

GO Significantly enrichment (padj < 0.05)

BP	MF	CC
glycerolipid biosynthetic process.	microtubule binding.	microtubule.
autophagy of peroxisome.	diacylglycerol O-acyltransferase activity.	phagophore assembly site.
photosynthesis.	fructose-bisphosphate aldolase activity.	cytoplasmic chromatin.
cell wall biogenesis.	DNA-binding transcription factor activity.	integral component of membrane.
xyloglucan metabolic process.	hydrolase activity, hydrolyzing O-glycosyl compounds.	plasma membrane.

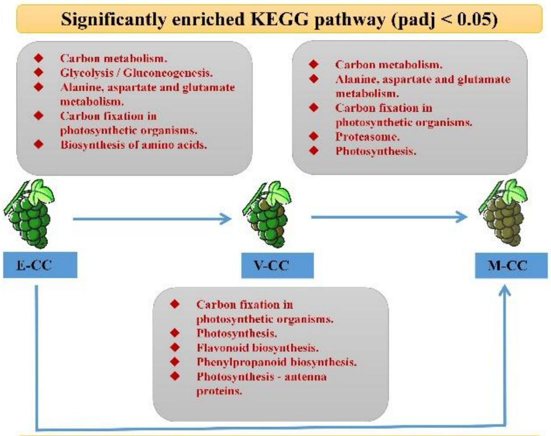

GO Significantly enrichment (padj < 0.05)

BP	MF	CC
xyloglucan metabolic process.	unfolded protein binding.	cytosol.
cellular iron ion homeostasis.	xyloglucan:xyloglucosyl transferase activity.	cytoplasm.
cellular zinc ion homeostasis.	transferase activity.	chloroplast thylakoid membrane.
cell wall biogenesis.	DNA-binding transcription factor activity.	photosystem II.
photosynthesis.	transferase activity, transferring acyl groups other than amino-acyl groups.	integral component of membrane.

Figure S3 The number of DEG in different developmental stages of grape fruit , numbers in red or black indicate the number of up- or down-regulated genes,

respectively, when compared and four other different ecological regions were significantly enriched in the top 5 KEGG vocabulary and at least two top 5 GOs in the three developmental periods.

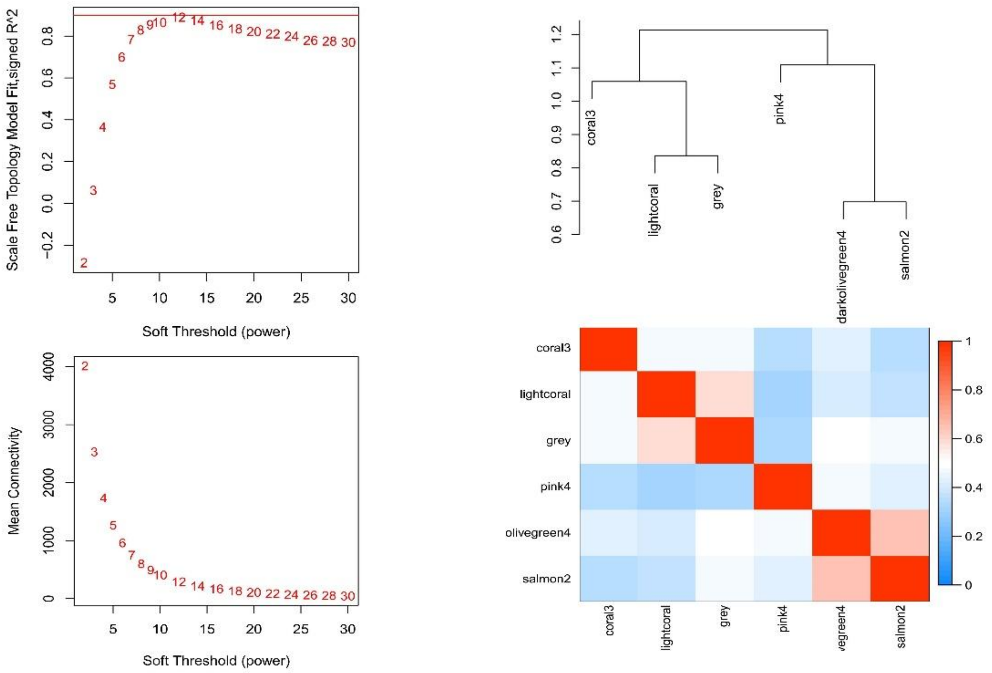

Figure S4 WGCNA scale-independence and average connectivity, transcriptome-trait correlations and cluster dendrogram and heatmap of correlations between modules with different traits.
